# A Rare Occurrence of Methicillin-Resistant Staphylococcus aureus (MRSA) Pneumonia Complicated by Nasal Vestibulitis and Orbital Cellulitis: A Case Report

**DOI:** 10.7759/cureus.63778

**Published:** 2024-07-03

**Authors:** Dhruv Kapoor, Shefali Gupta, Arijit Jotdar, Sankalp LNU

**Affiliations:** 1 Department of Otorhinolaryngology and Head and Neck Surgery, All India Institute of Medical Sciences, Raebareli, Raebareli, IND; 2 Department of Microbiology, All India Institute of Medical Sciences, Raebareli, Raebareli, IND; 3 Department of Cardio Thoracic and Vascular Surgery, All India Institute of Medical Sciences, Raebareli, Raebareli, IND

**Keywords:** preseptal orbital cellulitis, mrsa carrier state, nasal vestibulitis, mrsa community acquired pneumonia, mrsa

## Abstract

We report the case of left lower lobe community-acquired methicillin-resistant *Staphylococcus aureus* (MRSA) pneumonia in an immunocompetent male in his 20s. His illness was complicated by the dramatic appearance of right nasal vestibulitis and right preseptal orbital cellulitis post-admission. The patient responded well to vancomycin and made a complete recovery. Community-acquired MRSA pneumonia in immunocompetent adults is a rare entity in India, and the combination with vestibulitis has not yet been reported. This hitherto unreported presentation sheds further light on the evolving pattern of MRSA infections in the community.

## Introduction

While it is widely recognized that methicillin-resistant *Staphylococcus aureus* (MRSA) is frequently known to cause healthcare-associated pneumonia, an increasing number of reported cases have additionally demonstrated that community-acquired pneumonia (CAP) may be attributed to MRSA [1].

As the incidence of MRSA CAP in immunocompetent patients rises worldwide, it still remains infrequent in India [2]. This is a report of a case of MRSA pneumonia along with nasal vestibulitis and preseptal orbital cellulitis. Only one such case has been reported so far, in an elderly male with chronic obstructive pulmonary disease (COPD) [3]. In our case, the patient was a healthy, young male with no obvious co-morbidities. It is difficult to ascertain the temporal order of infection, although an MRSA carrier state with furunculosis leading to lung infection appears likely.

## Case presentation

A male in his early 20s, with no known co-morbidities, presented to the outpatient department with severe left-sided pleuritic chest pain for the past two days, accompanied by fever and shortness of breath. There was no history of recent hospitalization, prior repeated sinopulmonary infection, intravenous drug abuse, malignancy, or unprotected sexual activity. He was a security guard, physically fit otherwise, with occasional alcohol intake and tobacco smoking. On examination, the patient had tachypnea and tachycardia and was normotensive. There were two furuncles in the vestibule of the right nostril with overlying inflammation of the ala, and decreased air entry in the left inframammary, infra-axillary, and infrascapular regions.

**Figure 1:**
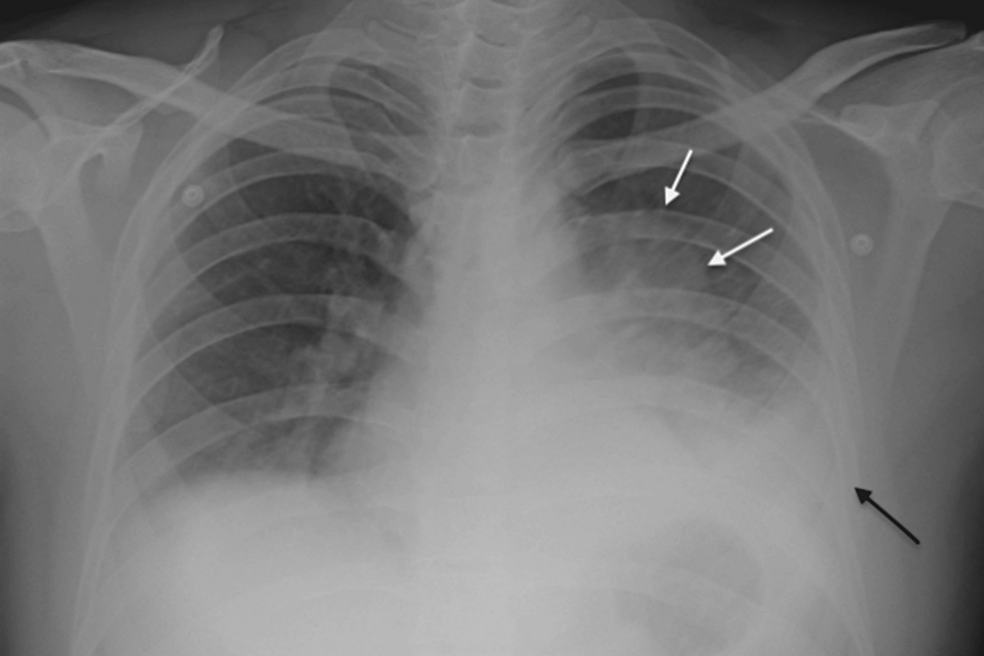
Chest radiograph showing blunting of left costophrenic angle (black arrow) with homogeneous radiopacity in the left middle and lower zone suggestive of left pleural effusion with underlying passive lung atelectasis (border marked by white arrows).

**Figure 2:**
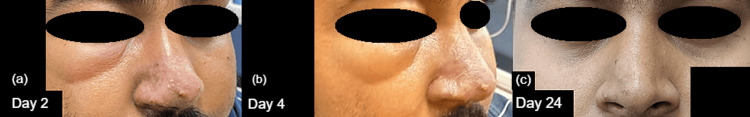
(a) On the second day of admission, the patient has swelling of the right upper and lower lids and right premaxillary region, along with inflammation at the tip of the nose; (b) On the fourth day of admission, the swelling around right upper and lower lids and right premaxillary regions have increased; (c) After 24 days of treatment, the swelling around the right eye, premaxillary region and tip of the nose is resolved.

Investigations revealed leukocytosis (24,210 cells/cc) with neutrophilia. The chest radiograph showed left lower lobe consolidation (Figure 1). He was empirically started on intravenous ceftriaxone and azithromycin. Over the next two days, the patient developed rapid swelling of the nose extending into the right cheek and orbital area (Figures 2a-2b). Further examination revealed a warm, tender, erythematous swelling over the right ala of the nose, causing a bulge in the nasal cavity. There was accompanying edema over the right cheek and right lower eyelid. Bilateral pupils were normal in size and reaction. There was no reduction in vision or limitation of eyeball movement, with no significant findings on ophthalmoscopy.

**Figure 3:**
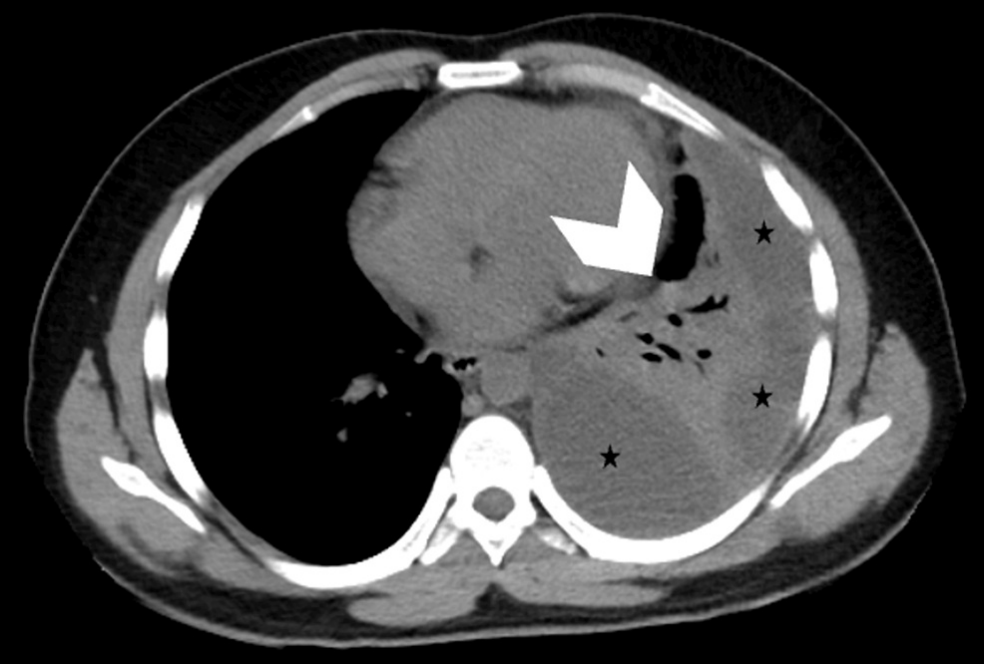
High-resolution computed tomography of the chest shows loculated pleural effusion (black stars) and right lower lung collapse (white arrowhead).

**Figure 4:**
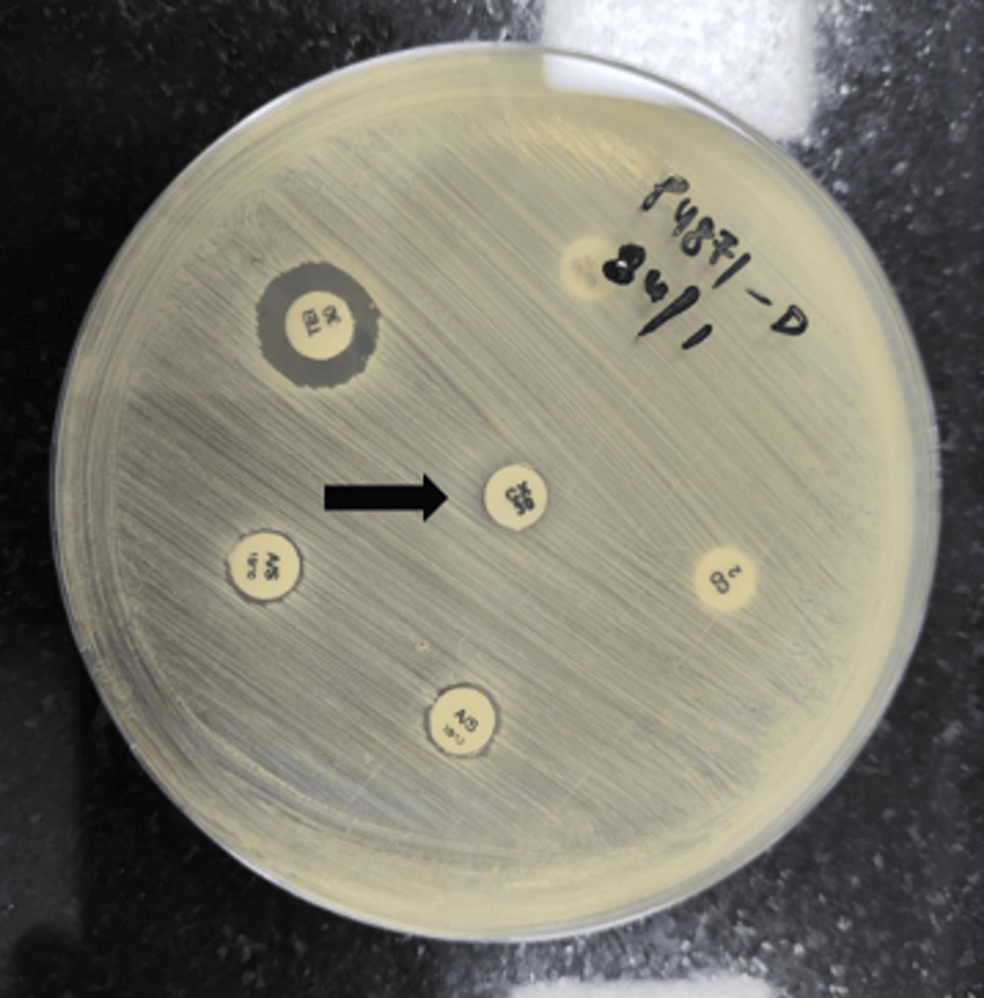
Kirby-Bauer’s disk diffusion method using Mueller-Hinton agar plate to determine antibiotic resistance. The black arrow points to the cefoxitin disc with no surrounding zone of inhibition. As per Clinical & Laboratory Standards Institute Guidelines, ≤23mm zone of inhibition around cefoxitin disc is considered a surrogate marker for MRSA. MRSA: methicillin-resistant *Staphylococcus aureus*

In view of swinging pyrexia and rapidly progressive facial swelling, antibiotics were empirically upgraded to vancomycin and piperacillin-tazobactam. Baseline and follow-up renal function tests were performed in view of the risk of nephrotoxicity. Ultrasound and high-resolution computed tomography (HRCT) chest revealed left lower lobe consolidation-collapse with loculated pleural effusion (Figure 3). Specimens of sputum, pleural fluid, blood, and urine were collected and sent for culture sensitivity as per standard guidelines. The methicillin resistance status of the test isolates was ascertained using the cefoxitin disc diffusion test (Figure 4).

Both pleural fluid and blood were positive for MRSA. Our isolates were sensitive only to vancomycin (MIC 1.5 µg/mL) and teicoplanin and resistant to erythromycin, clindamycin, tetracycline, gentamicin, ciprofloxacin, rifampin, and trimethoprim/sulphamethoxazole. Vancomycin and piperacillin-tazobactam were continued for two weeks. No clinical evidence of cavernous sinus thrombosis was noted. The patient’s fever and facial swelling completely subsided by the time of discharge. At the follow-up visit 10 days after discharge (Figure 2c), he was afebrile and pain-free, and his chest radiograph showed resolving pneumonia. He was counseled regarding MRSA carrier status and prescribed treatment for eradication of MRSA for himself and for the family members living with him (topical mupirocin ointment and chlorhexidine bath).

## Discussion

This is a case of MRSA CAP with nasal vestibulitis/preseptal orbital cellulitis in an immunocompetent young adult male with no known co-morbidities or predisposing factors. To the best of the authors’ knowledge, this is the first documented case from India.

According to recent Indian studies on MRSA infections, MRSA is not a common cause of CAP in India [4,5]. Nonetheless, there has been a rise in its occurrence, with a worldwide incidence rate of MRSA CAP standing at 3.1%. The rate remains comparatively low in India, at 1.4% [6]. Of Staphylococcal CAP, MRSA is responsible for 30-60% of cases [7].

Risk factors for MRSA CAP in patients include elderly status, chronic lung or kidney disease, increased regional resistance to antibiotics, prior MRSA infections, someone in the family with multidrug-resistant (MDR) pathogens, immunosuppression, and recurrent skin infections [4]. Laifer et al. reported an elderly male diagnosed with COPD who developed necrotizing pneumonia (MRSA CAP) after a nasal furuncle [3]. No such risk factors were present in our patient.

Recent literature shows evidence that nasal screening for MRSA helps assess the need for de-escalating the use of MRSA treatment. The test has a high negative predictive value but a low positive predictive value [8-10]. MRSA is more likely to induce complications in the form of orbital cellulitis. A limitation of our study is that we did not collect nasal swabs in a timely manner to confirm carrier status. In 2005, the U.S. Centres for Disease Control and Prevention (CDC) Active Bacterial Core (ABC) surveillance sites developed a standard description of community-associated MRSA (CA-MRSA) infections. CA-MRSA infections were defined as MRSA infections that have been identified in a patient's culture within 48 hours of hospital admission or in an outpatient setting [11,12]. Although this definition was adopted for this case, a strain-based definition of CA-MRSA is now preferred due to its distinct genetic profile, antibiotic resistance pattern, presentation, epidemiology, and therapy [13]. Because of limited resources, molecular epidemiological studies as in SCCmec typing and Panton-Valentine Leukocidin (PVL) gene detection could not be performed.

In this particular case, two facets require heightened emphasis. At first, the external nares served as a reservoir for *S. aureus*, including MRSA strains. Additionally, MRSA has been shown to result in CAP. This case study exhibits notable strength in its uncommon presentation, which holds significance in clinical settings and broader community-based contexts. A timely MRSA cover was given because pleural effusion is found consistently in Indian studies [4,5]. The patient had mild to moderate CAP and successfully recovered due to his immunocompetence.

## Conclusions

This case report is essential as this may reflect a shift in the behavior of pathogens in the community. It sheds light on the role of the nasal carrier state and lower respiratory infections, their complex interplay, and the possible threat of invasive disease posed by the MRSA carrier state, hitherto considered benign. Clinicians and microbiologists must be alert regarding the possibility of such infections becoming common.
